# Data in support of UbSRD: The Ubiquitin Structural Relational Database

**DOI:** 10.1016/j.dib.2015.10.007

**Published:** 2015-10-19

**Authors:** Joseph S. Harrison, Tim M. Jacobs, Kevin Houlihan, Koenraad Van Doorslaer, Brian Kuhlman

**Affiliations:** a Department of Biochemistry & Biophysics, University of North Carolina at Chapel Hill, Chapel Hill, NC 27599, United States; b Lineberger Comprehensive Cancer Center, Univer sity of North Carolina at Chapel Hill, Chapel Hill, NC 27599, USA; c DNA Tumor Virus Section, Laboratory of Viral Diseases, National Institute of Allergy and Infectious Diseases, National Institutes of Health, Bethesda, MD 209892, United States

## Abstract

This article provides information to support the database article titled “UbSRD: The Ubiquitin Structural Relational Database” (Harrison et al., 2015) [1] . The ubiquitin-like homology fold (UBL) represents a large family that encompasses both post-translational modifications, like ubiquitin (UBQ) and SUMO, and functional domains on many biologically important proteins like Parkin, UHRF1 (ubiquitin-like with PDB and RING finger domains-1), and Usp7 (ubiquitin-specific protease-7) (Zhang et al., 2015; Rothbart et al., 2013; Burroughs et al., 2012; Wauer et al., 2015) [2], [3], [4], [5]. The UBL domain can participate in several unique protein–protein interactions (PPI) since protein adducts can be attached to and removed from amino groups of lysine side chains and the N-terminus of proteins. Given the biological significance of UBL domains, many have been characterized with high-resolution techniques, and for UBQ and SUMO, many protein complexes have been characterized. We identified all the UBL domains in the PDB and created a relational database called UbSRD (Ubiquitin Structural Relational Database) by using structural analysis tools in the Rosetta (Leaver et al., 2013; O’Meara et al., 2015; Leaver-fay et al., 2011) [1], [6], [7], [8]. Querying UbSRD permitted us to report many quantitative properties of UBQ and SUMO recognition at different types interfaces (noncovalent: NC, conjugated: CJ, and deubiquitanse: DB). In this data article, we report the average number of non-UBL neighbors, secondary structure of interacting motifs, and the type of inter-molecular hydrogen bonds for each residue of UBQ and SUMO. Additionally, we used PROMALS3D to generate a multiple sequence alignment used to construct a phylogram for the entire set of UBLs (Pei and Grishin, 2014) [9]. The data described here will be generally useful to scientists studying the molecular basis for recognition of UBQ or SUMO.

**Specifications Table**

**Table:** 

Subject area	*Bioinformatics and Biology*
More specific subject area	*Ubiquitin-like homology domain structural biology*
Type of data	*Histograms of per residue properties for UBQ and SUMO, phylogenetic clustering, and UBL schematic.*
How data was acquired	*Computational analysis of protein structures using the Rosetta features analysis protocol*
Data format	*Figures and sqlite*3 *database*
Experimental factors	*Rosetta*3 *features analysis of renumbered PDBs*
Experimental features	*We identified all the UBL-containing structures in the PDB, grouped them by type of PPI, and used structural classification tools in Rosetta to quantify measurable properties of these structures.*
Data source location	*University of North Carolina*
Data accessibility	*http://rosettadesign.med.unc.edu/ubsrd/*

**Value of the data:**

• A description of how we created UbSRD that can be used as a template for researching wishing to construct a Rosetta features database.
• Presents phylogenetic clustering for the ubiquitin homology folds.
• Reports per residue statistics of the molecular properties of UBQ and SUMO participating in protein–protein interactions, generally useful for researchers investigating proteins that recognize UBQ and SUMO.

## 1 Data experimental design, materials and methods

### 1.1 Experimental design

#### 1.1.1 Identifying ubiquitin-homology domains in the PDB and constructing an Rosetta features SQL database

**Fig. 1:**
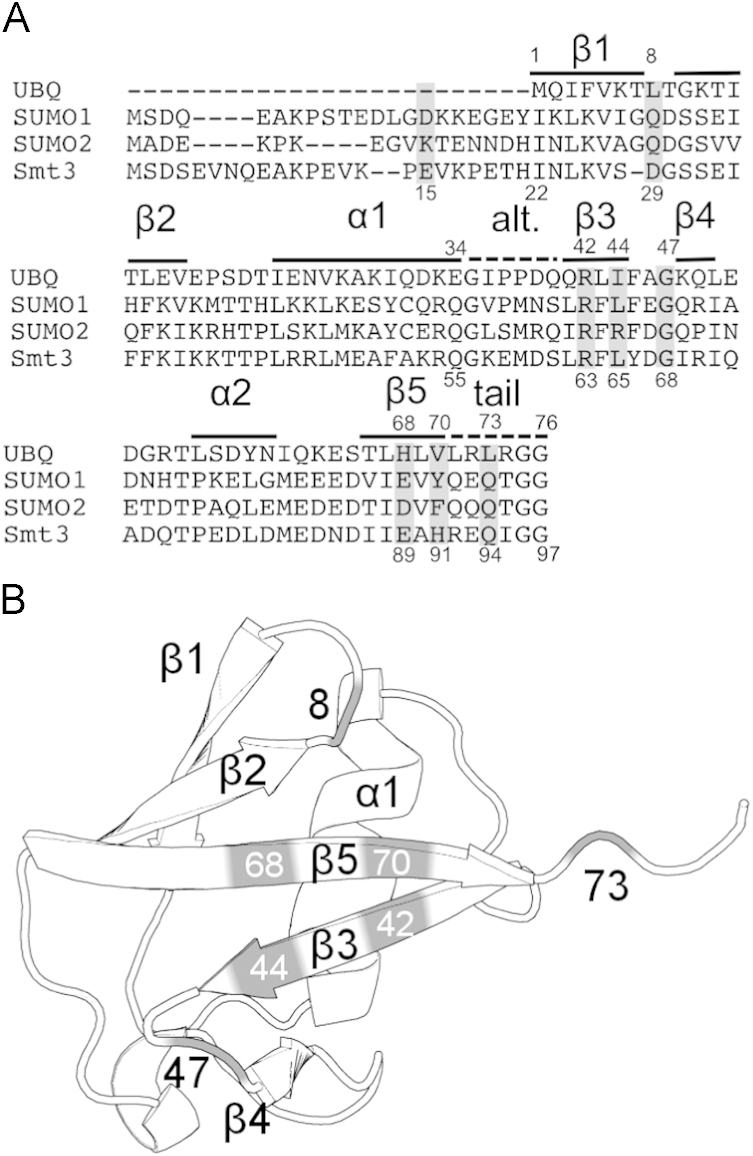
(A) Sequence alignment between ubiquitin (UBQ), SUMO1, SUMO2 and SMT3, the *S. cerevisiae* SUMO homolog. (B) Cartoon representation of the ubiquitin-like homology fold (UBL) with secondary structure elements annotated.

**Fig. 2:**
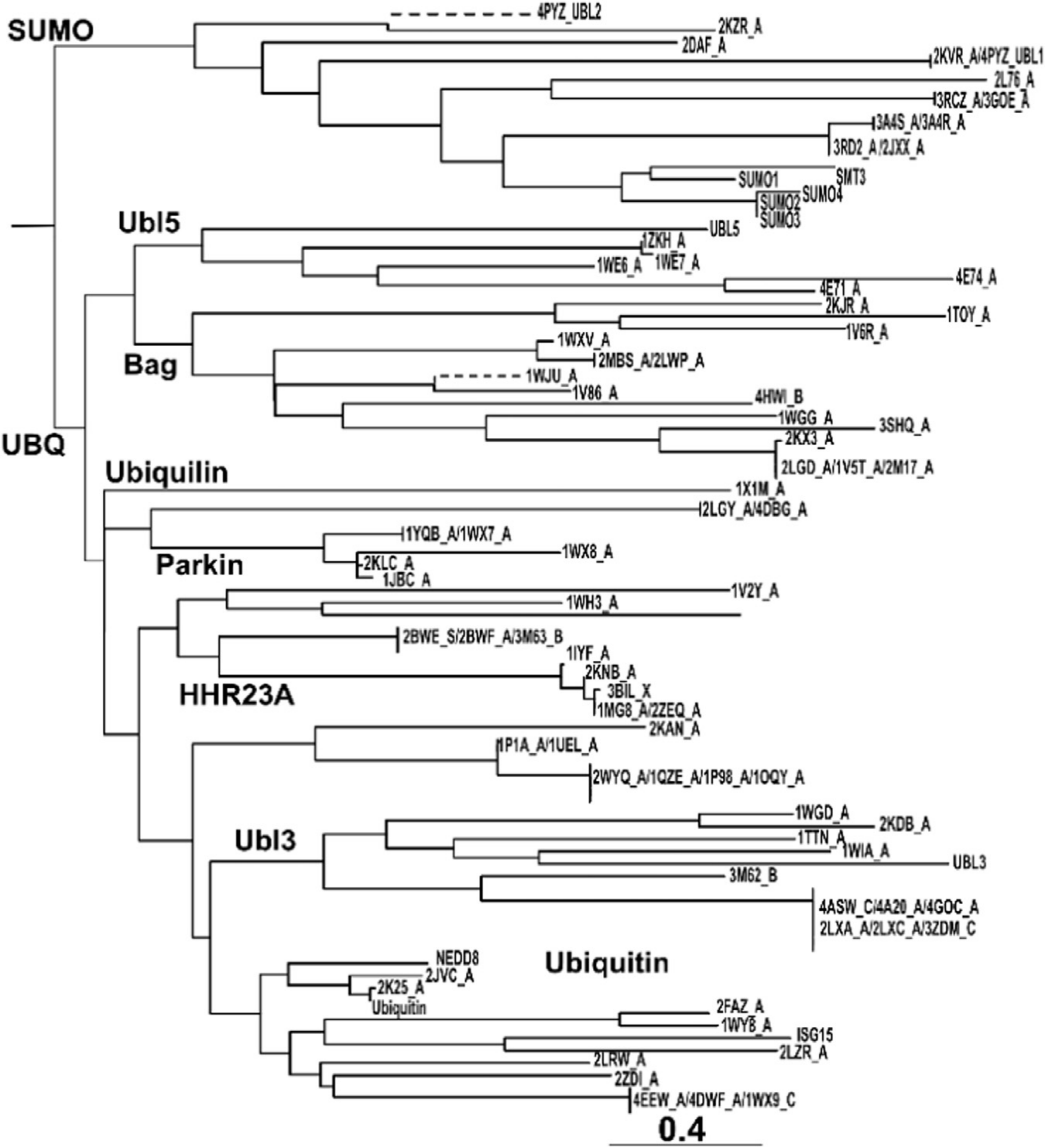
(A) Phylogenetic clustering of UBLs in UbSRD, dashed lines indicate longer branches (see [1] for methods). An expanded version of the phylogram can be found at http://rosettadesign.med.unc.edu/ubsrd/#browse/phylogeny.

**Fig. 3:**
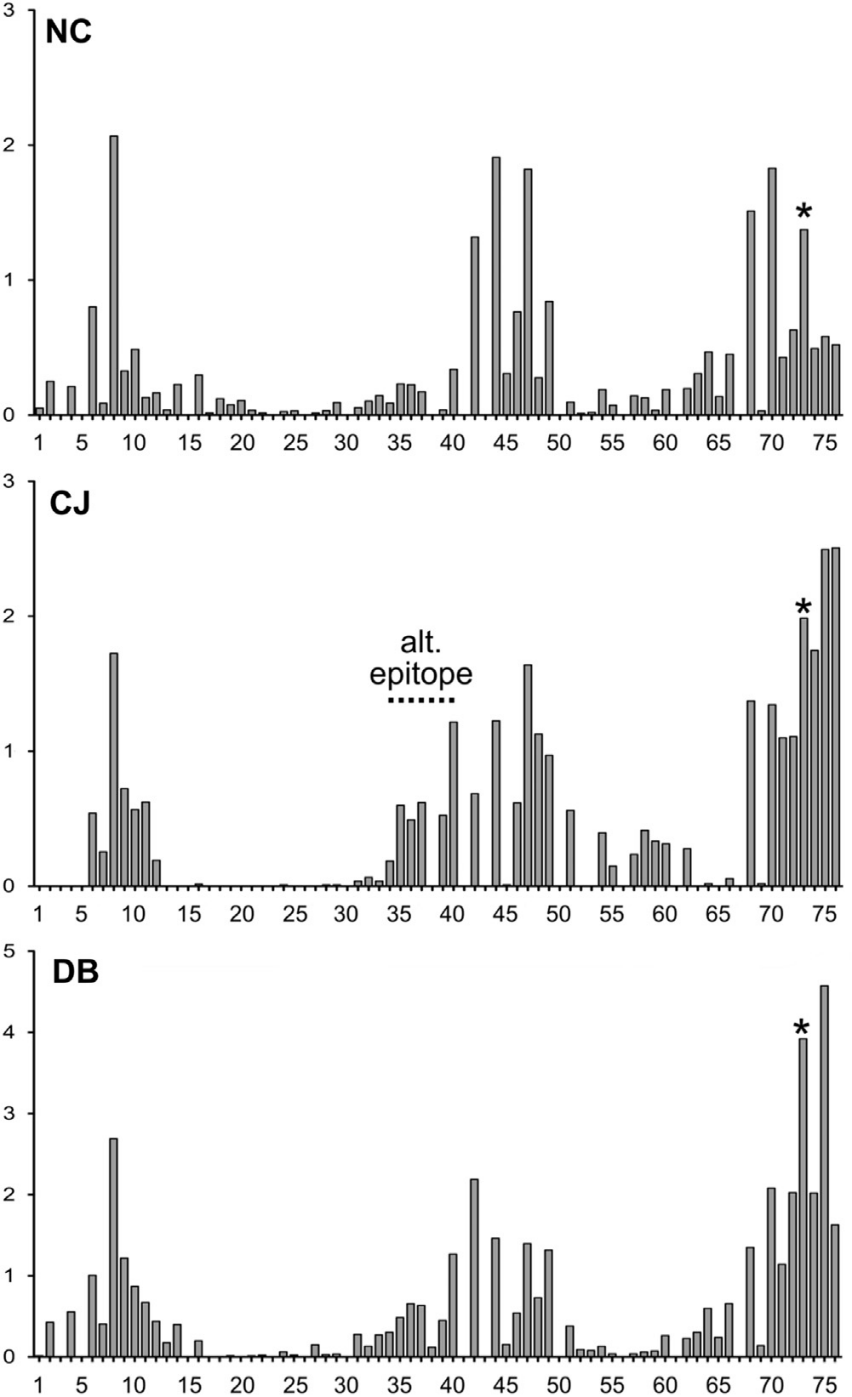
Number of non-UBQ amino acid neighbors for each residue of UBQ using a 6 Å distance cutoff. The ubiquitin structures are grouped by the following protein–protein interactions: NC: noncovalent, CJ: conjugated, DB: deubiquitinase. The units on the *Y*-axis are average number of normalized non-UBQ neighboring residues per PDB.

**Fig. 4:**
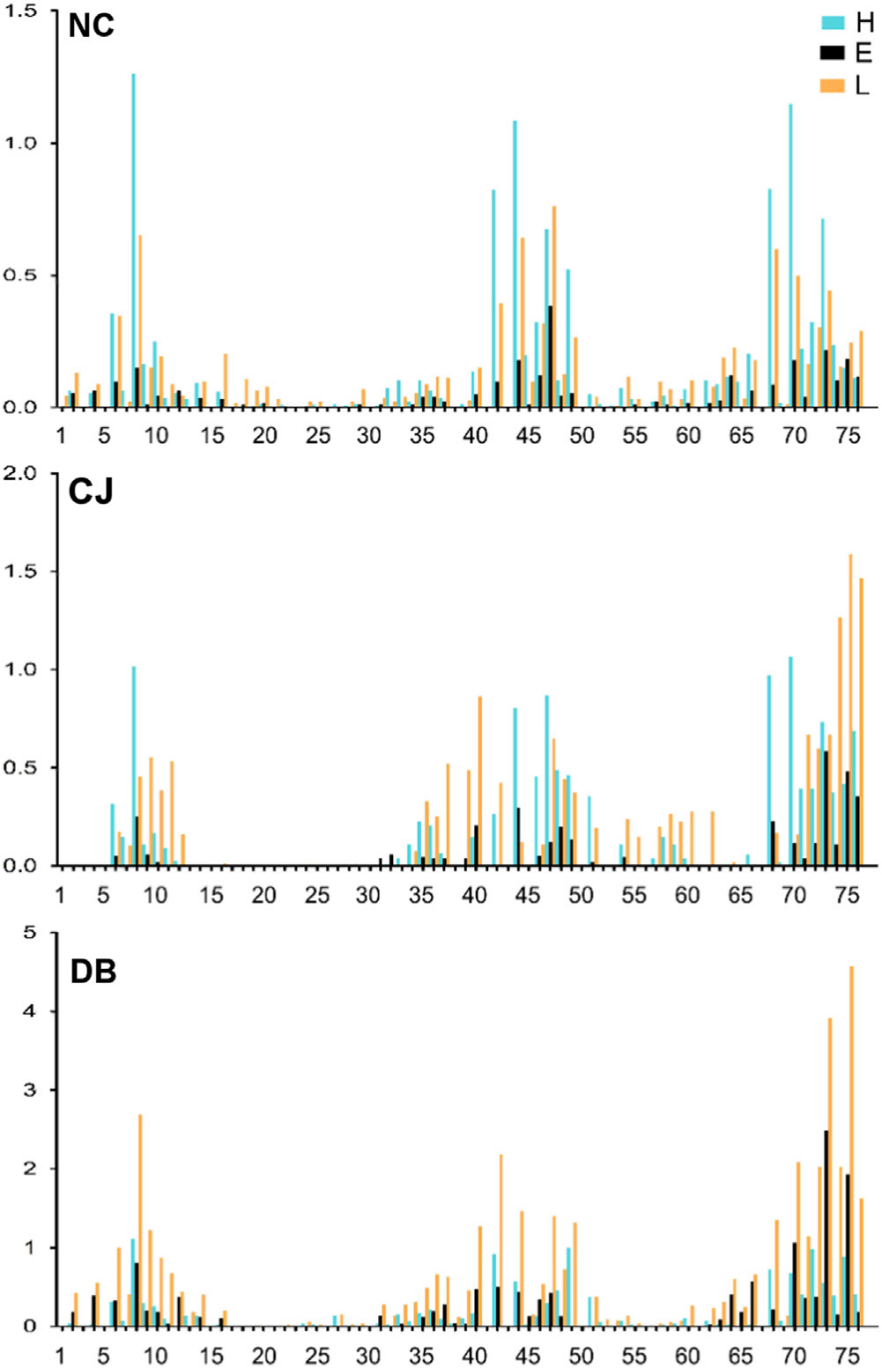
Secondary structure of UBQ interacting motifs for each residue of UBQ. We classified secondary structure using the simplified DSSP distinction, *H α-helix*, *E β-strand*, *L loop*. The ubiquitin structures are grouped by the following protein–protein interactions: NC: noncovalent, CJ: conjugated, DB deubiquitinase. The *Y*-axis represents the average number of normalized interacting from each secondary structure type per PDB.

**Fig. 5:**
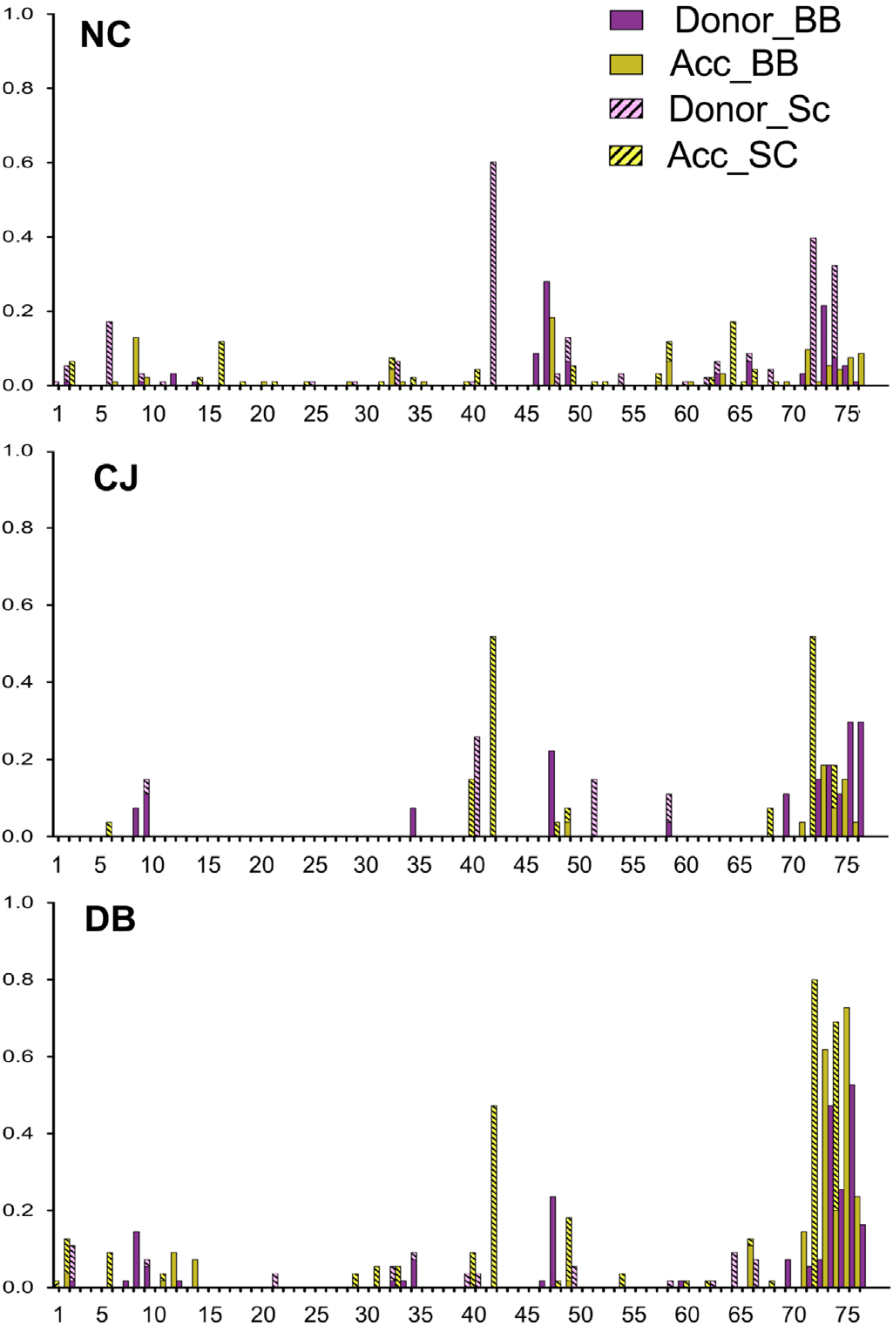
Inter-molecular hydrogen bond sites on UBQ. We detected hydrogen bonds using Rosetta hydrogen bond score. Each hydrogen bond was classified as either a donor or acceptor and if the chemical moiety participating in the hydrogen bond belongs to the peptide backbone or the side chain. The ubiquitin structures are grouped by the following protein–protein interactions: NC: noncovalent, CJ: conjugated, DB: deubiquitinase. The *Y*-axis represents average number of hydrogen bonds per PDB. Redundant hydrogen bonds in structure containing multiple ubiquitin chains were only counted once per PDB.

**Fig. 6:**
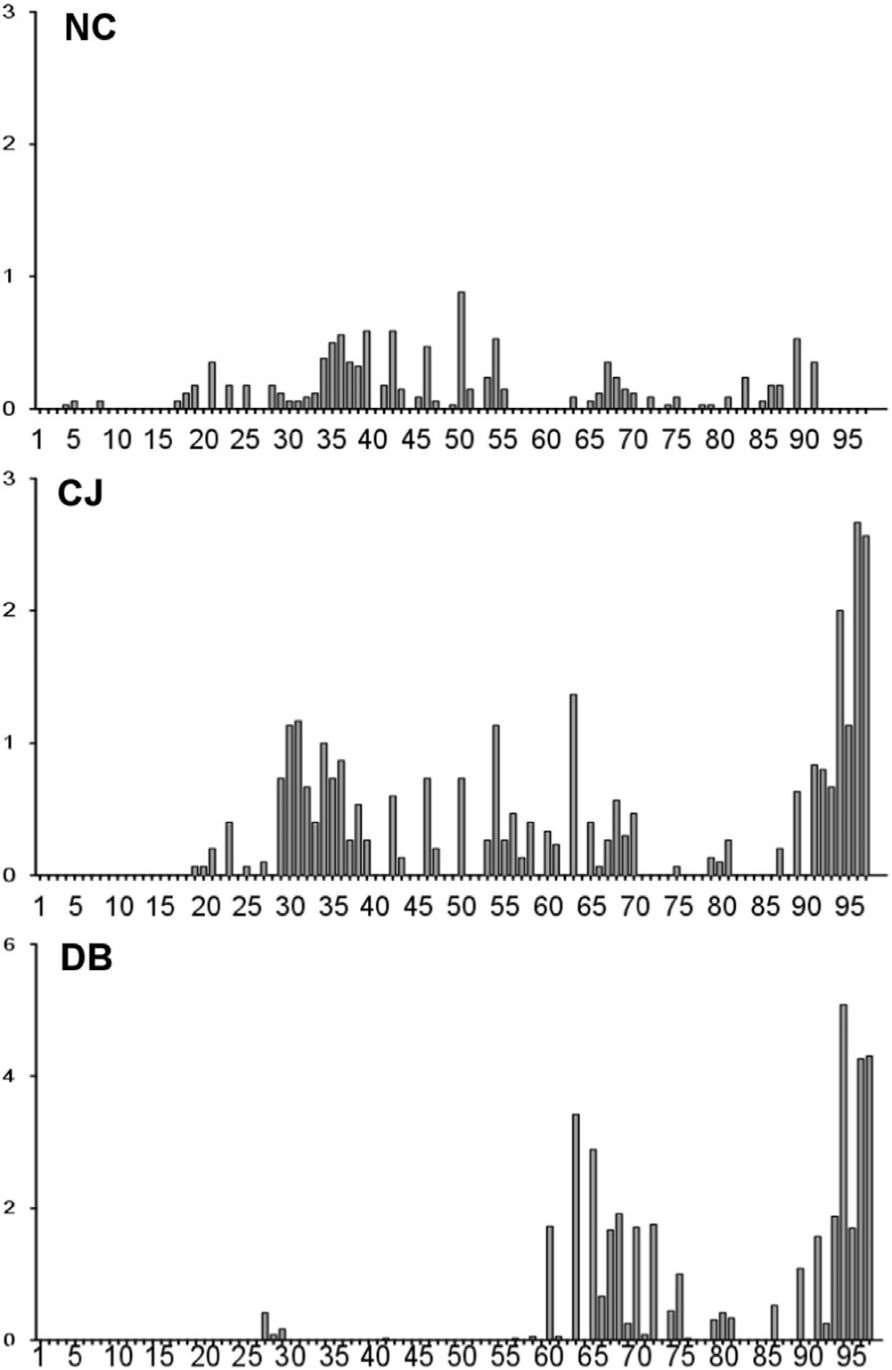
Number of non-SUMO amino acid neighbors for each residue of SUMO using a 6 Å distance cutoff. The ubiquitin structures are grouped by the following protein*–*protein interactions: NC: noncovalent, CJ: conjugated, DB: deubiquitinase. The units on the *Y*-axis are average number of normalized non-UBQ neighboring residues per PDB. The SUMO1 numbering scheme is used for all SUMO molecules (Fig. 1).

**Fig. 7:**
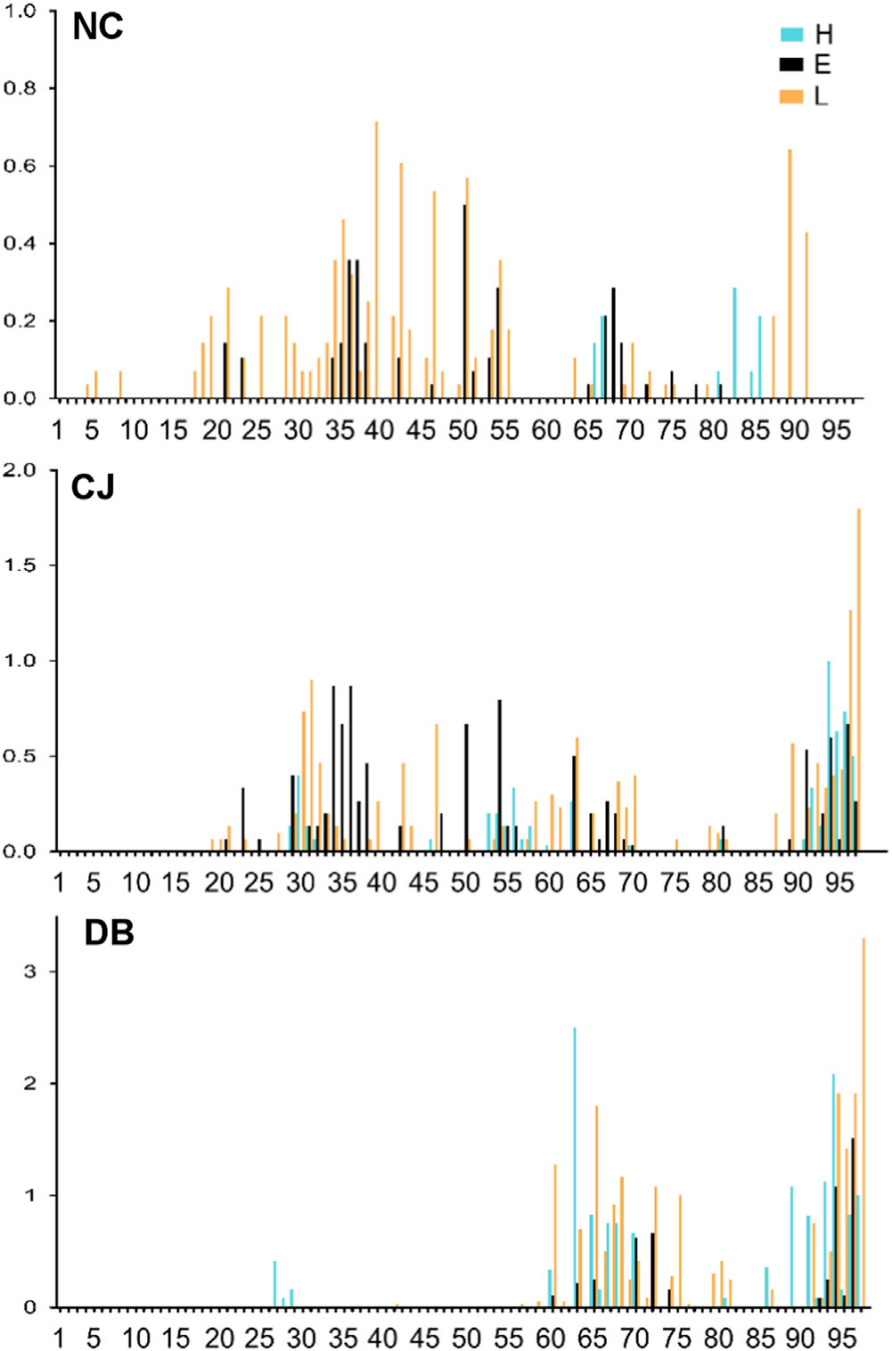
Secondary structure of SUMO interacting motifs. The secondary structure was determined using the following simplified DSSP distinction, *H α-helix*, *E β-strand*, *L loop*. The SUMO structures are grouped by the following protein–protein interactions: NC: noncovalent, CJ: conjugated, DB: deubiquitinase. The *Y*-axis represents the average number of normalized interacting residues in each secondary structure element per PDB. The SUMO1 numbering scheme is used for all SUMO molecules (Fig. 1).

**Fig. 8:**
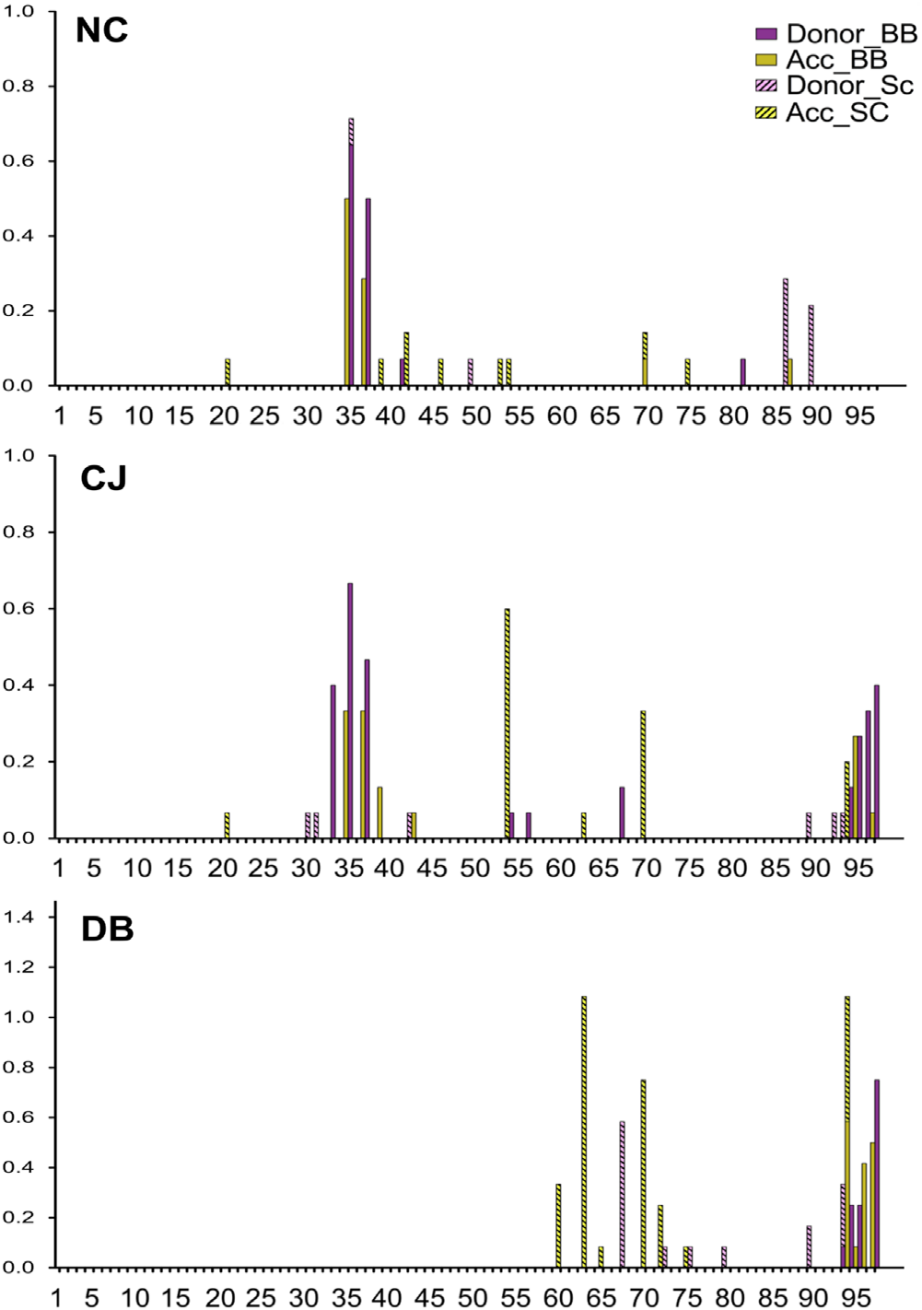
Inter-molecular hydrogen bond sites on SUMO. We detected hydrogen bonds using Rosetta hydrogen bond score. Each hydrogen bond was classified as either a donor or acceptor and if the chemical moiety participating in the hydrogen bond belongs to the peptide backbone or the side chain. The SUMO structures are grouped by the following protein–protein interactions: NC: noncovalent, CJ: conjugated, DB: deubiquitinase. The *Y*-axis represents average number of hydrogen bonds per PDB. The SUMO1 numbering scheme is used for all SUMO molecules (Fig. 1). Redundant hydrogen bonds in structure containing multiple SUMO chains were only counted once per PDB.

To identify all the all the UBL domains in the PDB, we used delta PSI-blast since the standard blast algorithm produced many false positives [10], [11]. Using the sequences of UBQ, SUMO and SMT3, the *S. cerevisiae* SUMO homolog, we performed seven iterative rounds of delta-psi blast, downloaded the hit table, and used a one line shell script { grep -o "pdb|\<….\>|" “hit_table_file_name” | cut -d׳|׳ -f2 | sort | uniq } to generate a list of PDB codes to run the Rosetta features analysis on [6]. The features analysis is invoked through the Rosetta Scripts interface and the executable, flags, and Rosetta script needed to run this analysis are found in Supporting file 1 [12]. This analysis will create and SQLite database of Rosetta derived features (for SQLite syntax see [13]) and the recorded features in UbSRD are listed in the Rosetta Script Supporting file 1. It is worth noting the importance of the “jd2:delete_old_poses” flag when running this analysis, otherwise each structure will be stored in memory using a lot of RAM. We manually categorized each structure by the type of UBL and PPI and then used a series of Python scripts to identify, renumber, and generate an SQL table of UBQ and SUMO chains [1]. We further classified each UBQ and SUMO chain by the type of PPI and for UBQ, the type of polymer. Each manually generated table was imported into the SQL database and the syntax for creating and importing tables into existing SQL databases is found in Supporting file 2. We employed a 6 Å distance cutoff from the action coordinate, the average geometric center of the side chain, as a criterion for selecting neighboring residues and the SQL query used to report the residue neighbors is found in Supporting file 3. To compute PDB averages for UBL recognition, each structure was normalized by the number of UBL chains participating in the same type of protein–protein interaction. Fig. 1, Fig. 2, Fig. 3, Fig. 4, Fig. 5, Fig. 6, Fig. 7, Fig. 8

## Appendix A Supplementary material

Supporting File 1: Rosetta executable, options, and Rosetta Script

Supporting File 2: Syntax for generating new tables in an SQL database

Supporting File 3: SQL query to select UBL neighbors
